# Effectiveness of gel-immersion endoscopy for examination for suspected pancreaticobiliary maljunction

**DOI:** 10.1055/a-2325-4853

**Published:** 2024-06-07

**Authors:** Koichi Soga, Takeshi Fujiwara, Fuki Hayakawa, Ikuhiro Kobori, Masaya Tamano

**Affiliations:** 1 26263 Department of Gastroenterology, Dokkyo Medical University Saitama Medical Center, Koshigaya, Japan

Pancreaticobiliary maljunction (PBM) is a congenital anomaly in which the pancreatic and bile ducts join outside the duodenal wall and pancreatic juices and bile flow into a single channel 1 . Endoscopic retrograde cholangiopancreatography (ERCP) clearly shows the connecting structures and is the most effective method for detecting PBM (sensitivity, 90%–100%) 2 . Here, we aim to show the effectiveness of gel-immersion endoscopy for diagnostic differentiation and/or examination of PBM.

A 43-year-old woman was referred to our hospital for examination of a suspected PBM. Gel-immersion endoscopic ultrasonography (GI-EUS) and GI-ERCP were performed. For gel-immersion endoscopy, an auxiliary injection cap (BioShield Irrigator; US Endoscopy, Mentor, Ohio, USA) was used to allow the operative channel to remain free, and Viscoclear gel (Otsuka Pharmaceutical Factory, Tokushima, Japan) was injected before and during endoscopy 3 . GI-EUS enables better observations of the duodenal ampulla with a relatively normal gastrointestinal environment compared to observations made using an underwater technique 4 . GI-EUS revealed a normal confluence between the bile duct and the pancreatic duct, ruling out PBM ( Fig. 1 ). Additionally, GI-ERCP revealed no bile duct irregularities, also ruling out PBM ( Fig. 2 ). Notably, GI-ERCP can be performed in a relatively normal gastrointestinal environment, with no overstressing of the intestinal tract or papillary region, such as occurs with air delivery or intestinal stretching. Sufficient contrast medium can be injected from the pancreaticobiliary junction to the duodenum ( Fig. 3 ), thereby improving the accuracy of the examination. Patients with a long common channel, in which communication between the pancreatic and bile ducts is maintained during relaxation and contraction of the sphincter under serial observations during ERCP, are diagnosed with PBM 5 . Gel-immersion endoscopic procedures allow lower levels of intraluminal pressure and maintenance of wall tension compared with those using gas insufflation. We believe that cholangiopancreatic examination using GI-EUS and GI-ERCP, which do not require insufflation of gas into the duodenum, is less stressful to the duodenal ampulla ( Video 1 ).

**Fig. 1:**
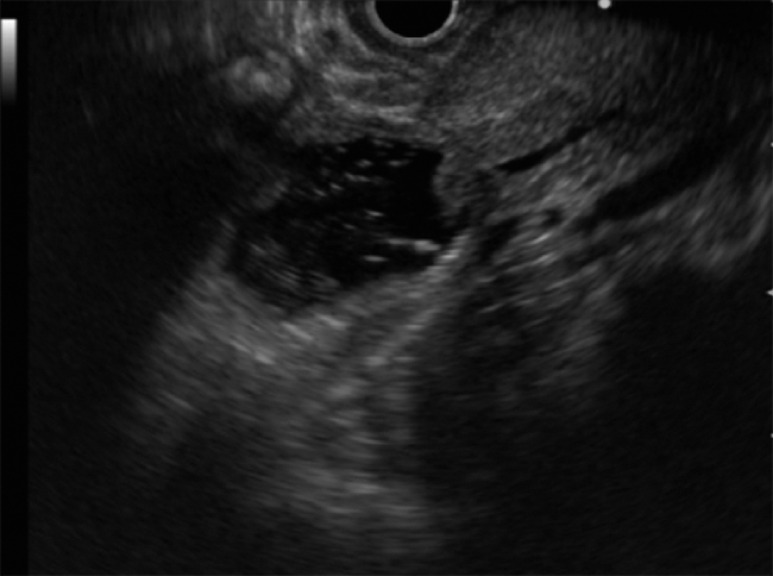
Gel-immersion endoscopic ultrasonography (GI-EUS) of a 43-year-old woman admitted for examination of suspected pancreaticobiliary maljunction. GI-EUS, like the underwater technique, provides excellent visualization of the duodenal ampulla. Here, GI-EUS shows a normal confluence between the pancreatic duct and bile duct, ruling out pancreaticobiliary maljunction.

**Fig. 2:**
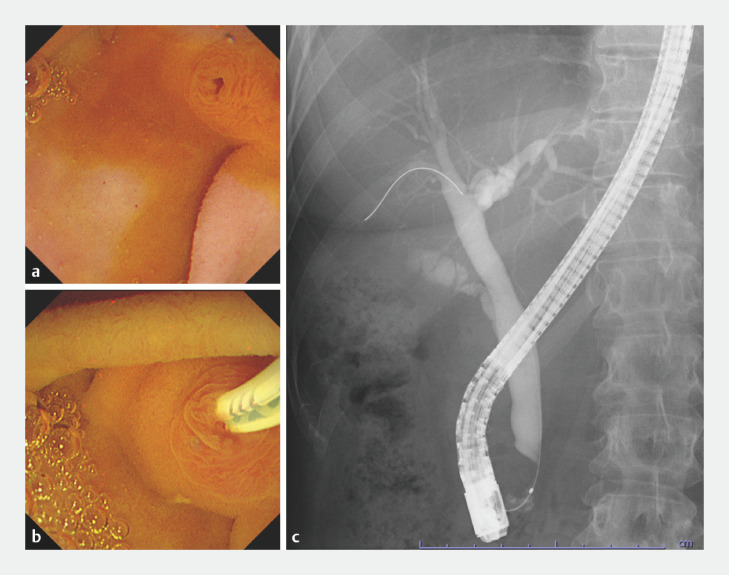
Gel-immersion endoscopic retrograde cholangiopancreatography (GI-ERCP) examination to screen for pancreaticobiliary maljunction. **a** The gel fills the duodenum; therefore, excessive load is not applied to the duodenal ampulla area, enabling observation of cases in which bile juice drains spontaneously into the duodenum. **b** The ERCP cannula is easily inserted into the common bile duct from the duodenal papilla. **c** Sufficient contrast medium can be injected from the pancreaticobiliary junction to the duodenum, leading to improved examination accuracy.

**Fig. 3:**
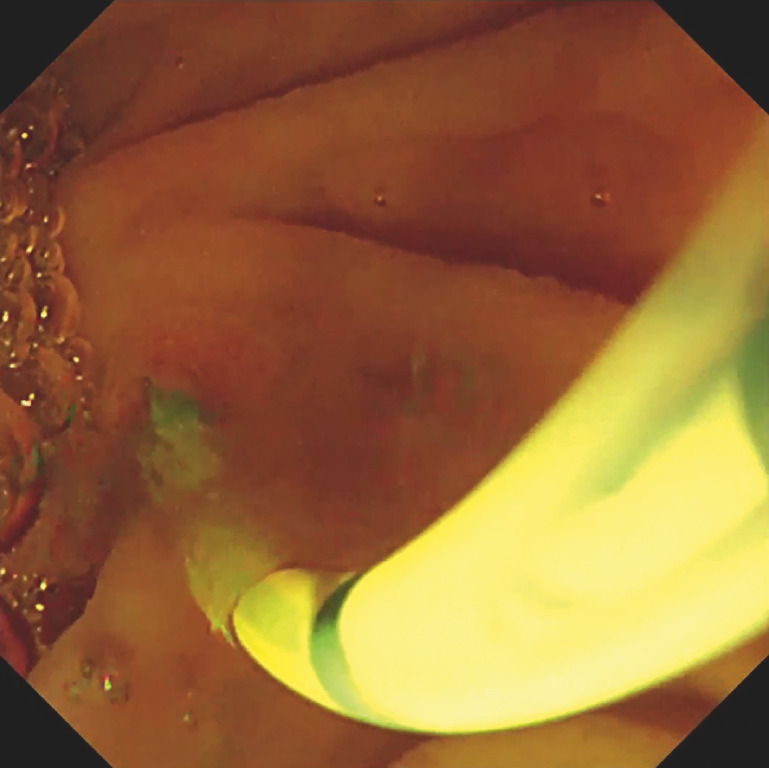
GI-ERCP can enable the clinician to confirm that sufficient contrast has passed from the bile duct to the duodenum. The endoscopic image confirms that sufficient contrast was injected from the cannula, based on the difference in osmotic pressure between the gel and the contrast medium.

Gel-immersion endoscopic ultrasonography and gel-immersion endoscopic retrograde cholangiopancreatography in a 43-year-old woman with suspected pancreaticobiliary maljunction. Pancreaticobiliary maljunction was ruled out using the two modalities.Video 1

Endoscopy_UCTN_Code_TTT_1AS_2AD

## Notes

Endoscopy E-Videos https://eref.thieme.de/e-videos
*E-Videos* is an open access online section of the journal *Endoscopy* , reporting on interesting cases and new techniques in gastroenterological endoscopy. All papers include a high-quality video and are published with a Creative Commons CC-BY license. Endoscopy E-Videos qualify for HINARI discounts and waivers and eligibility is automatically checked during the submission process. We grant 100% waivers to articles whose corresponding authors are based in Group A countries and 50% waivers to those who are based in Group B countries as classified by Research4Life (see: https://www.research4life.org/access/eligibility/ ). This section has its own submission website at https://mc.manuscriptcentral.com/e-videos .
